# Lp-PLA2 levels in HIV-infected patients

**DOI:** 10.7448/IAS.17.4.19721

**Published:** 2014-11-02

**Authors:** Beatriz Díaz-Pollán, Vicente Estrada, Manuel Fuentes-Ferrer, Dulcenombre Gómez-Garré, Jesús San Román-Montero

**Affiliations:** 1 Medicina Interna, Hospital Universitario La Paz, Madrid, Spain; 2 Medicina Interna III, Hospital Clínico San Carlos, Madrid, Spain; 3 Preventive Medicine Department, Hospital Clínico San Carlos, , Madrid, Spain; 4 Vascular Biology Research Laboratory, Instituto de Investigación Sanitaria del Hospital Clínico San Carlos (IdISSC), Madrid, Spain; 5 Departamento de Medicina y Cirugía, Universidad Rey Juan Carlos, Alcorcón, Spain

## Abstract

**Introduction:**

HIV-infected patients show an increased risk of cardiovascular disease (CVD). In the general population, lipoprotein-associated phospholipase A2 (Lp-PLA2) appears to be an independent predictor of CVD. We aimed to study associations between Lp-PLA2 plasma levels and other risk factors for CVD in HIV patients.

**Materials and Methods:**

A cross-sectional, comparative study of two series of cases (HIV patients, n=116 and age-matched non-HIV healthy controls, n=113) was conducted. Eighty-seven percent HIV patients on antiretroviral therapy (ART), 72.4% with HIV-1 viral load <50 cop/mL. Inflammatory biomarkers (CRP, Lp-PLA2) and internal carotid intima-media thickness (IMT) were measured and CVD risk (Framingham and SCORE algorithms) was calculated. Univariate and multivariable associations between these variables were performed.

**Results:**

HIV patients presented higher Lp-PLA2 levels [276.81 ng/mL (209.71–356.58)] than uninfected healthy controls [220.80 ng/mL (172.70–256.90)], p≤0.01. In univariate analysis of the global sample, only cigarette smoking was associated with higher Lp-PLA2 levels, p≤0.001. In HIV group, female and smoker patients showed higher Lp-PLA2 levels, p≤0.05. No significant association was found between Lp-PLA2 levels and another CVD risk factors, carotid IMT, Framingham and SCORE algorithms, ART, HIV-1 viral load neither and CD4+ T lymphocyte count. In multivariate analysis, cigarette smoking remained significantly associated with Lp-PLA2 levels [β=64.8 (95% CI 10.8–118.9) ng/mL, p=0.020].

**Conclusions:**

HIV-infected patients present higher Lp-PLA2 levels than healthy controls, and in this population, tobacco smoking is significantly associated with increased Lp-PLA2 levels. Smoking cessation should be a priority in CVD prevention in HIV-infected patients.

